# Chat Generative Pre-Trained Transformer (ChatGPT) in Oral and Maxillofacial Surgery: A Narrative Review on Its Research Applications and Limitations

**DOI:** 10.3390/jcm14041363

**Published:** 2025-02-18

**Authors:** Sung-Woon On, Seoung-Won Cho, Sang-Yoon Park, Ji-Won Ha, Sang-Min Yi, In-Young Park, Soo-Hwan Byun, Byoung-Eun Yang

**Affiliations:** 1 Division of Oral and Maxillofacial Surgery, Department of Dentistry, Dongtan Sacred Heart Hospital, Hallym University College of Medicine, Hwaseong 18450, Republic of Korea; drummer0908@hanmail.net (S.-W.O.); hajiwon@hallym.or.kr (J.-W.H.); 2 Department of Artificial Intelligence and Robotics in Dentistry, Graduated School of Clinical Dentistry, Hallym University, Chuncheon 24252, Republic of Korea; kotneicho@gmail.com (S.-W.C.); psypjy0112@naver.com (S.-Y.P.); queen21c@hallym.or.kr (S.-M.Y.); denti2875@hallym.or.kr (I.-Y.P.); purheit@daum.net (S.-H.B.); 3 Institute of Clinical Dentistry, Hallym University, Chuncheon 24252, Republic of Korea; 4 Department of Oral and Maxillofacial Surgery, Hallym University Sacred Heart Hospital, Anyang 14066, Republic of Korea; 5 Dental Artificial Intelligence and Robotics R&D Center, Hallym University Medical Center, Anyang 14066, Republic of Korea; 6 Department of Orthodontics, Hallym University Sacred Heart Hospital, Anyang 14066, Republic of Korea

**Keywords:** generative artificial intelligence, ChatGPT, chatbot, surgery, oral, oral and maxillofacial surgery, review literature as topic

## Abstract

**Objectives:** This review aimed to evaluate the role of ChatGPT in original research articles within the field of oral and maxillofacial surgery (OMS), focusing on its applications, limitations, and future directions. **Methods**: A literature search was conducted in PubMed using predefined search terms and Boolean operators to identify original research articles utilizing ChatGPT published up to October 2024. The selection process involved screening studies based on their relevance to OMS and ChatGPT applications, with 26 articles meeting the final inclusion criteria. **Results**: ChatGPT has been applied in various OMS-related domains, including clinical decision support in real and virtual scenarios, patient and practitioner education, scientific writing and referencing, and its ability to answer licensing exam questions. As a clinical decision support tool, ChatGPT demonstrated moderate accuracy (approximately 70–80%). It showed moderate to high accuracy (up to 90%) in providing patient guidance and information. However, its reliability remains inconsistent across different applications, necessitating further evaluation. **Conclusions**: While ChatGPT presents potential benefits in OMS, particularly in supporting clinical decisions and improving access to medical information, it should not be regarded as a substitute for clinicians and must be used as an adjunct tool. Further validation studies and technological refinements are required to enhance its reliability and effectiveness in clinical and research settings.

## 1. Introduction

Generative artificial intelligence (AI) has rapidly advanced over the past three to four years, gaining widespread attention and adoption. This subset of AI focuses on content creation rather than mere data analysis, enabling the generation of text, images, and other media in response to user prompts [1]. A significant driver of this progress has been the development of transformer-based artificial neural networks, particularly large language models (LLMs) [2]. Among these, Chat Generative Pre-Trained Transformer (ChatGPT), Copilot (formerly Bing Chat Enterprise), Gemini (formerly Bard), and Large Language Model Meta AI (Llama) represent the most well-known AI-driven conversational agents. ChatGPT, in particular, has emerged as the most widely recognized and utilized generative AI system, surpassing the usage rates of Gemini and Copilot by two to three times [3].

First launched on 30 November 2022, ChatGPT reached over 100 million users by 2023 [4]. Initially based on GPT-3.5, it was fine-tuned for conversational applications through Reinforcement Learning from Human Feedback (RLHF), which enhanced its ability to align responses with human preferences while reducing biases [5]. The continuous refinement of ChatGPT has led to substantial improvements in natural language processing, training datasets, and model architecture. In 2023, GPT-4 introduced enhanced accuracy, contextual awareness, and reasoning capabilities, alongside multimodal functions enabling text and image processing [6]. Compared to GPT-3.5, GPT-4 is more reliable, less error prone, and better equipped to follow complex instructions [6]. The release of GPT-4o on 13 May 2024 further expanded these capabilities, improving accessibility for free-tier users. By 29 August 2024, ChatGPT had amassed 200 million weekly active users [7].

Given its rapid evolution and growing popularity, ChatGPT is increasingly applied in healthcare and medicine, encompassing education, clinical practice, and research [8,9]. In medical education, ChatGPT enhances personalized learning for students in medicine, dentistry, and pharmacy by providing interactive content, step-by-step guidance, and real-time feedback on clinical techniques [9]. These features are particularly valuable for understanding complex medical concepts and facilitating problem-based learning. In clinical practice, ChatGPT assists in medical documentation, patient note summarization, and patient education by simplifying medical jargon to improve health literacy [8]. Additionally, it streamlines workflows by supporting clinicians in administrative tasks. ChatGPT facilitates literature reviews, data analysis, and code generation for experiments in research, accelerating scientific inquiry and drug discovery [8]. Its role in enhancing scientific writing and supporting systematic reviews further underscores its value to the medical community.

Oral and maxillofacial surgery (OMS) is a highly specialized discipline that integrates medicine and dentistry to address conditions affecting the oral cavity, jaws, and facial structures [10]. This field demands expertise in craniofacial anatomy, oral pathology, surgical techniques, and anesthesia management. OMS procedures range from corrective jaw surgeries and dental implant placements to complex reconstructive surgeries following trauma or oncologic resections [11]. OMS relies on advanced diagnostic tools and evidence-based treatment approaches because it overlaps with related fields such as plastic surgery, otolaryngology, and neurology [12]. The complexity of OMS cases necessitates precise treatment planning, where AI-driven tools like ChatGPT can offer significant support. By analyzing extensive patient data—including medical records, imaging studies, and clinical documentation—ChatGPT has the potential to assist in surgical decision making and treatment optimization [13].

Despite ChatGPT’s increasing integration into various medical fields, there remains a lack of comprehensive reviews examining its role in OMS research. Given its diverse applications in this domain, particularly in generating ideas for systematic reviews and supporting research workflows, a thorough investigation is warranted [14].

This review aims to address this gap by analyzing the applications of ChatGPT in original OMS research articles, evaluating its current limitations, and exploring future directions for its implementation in this field.

## 2. Methods

A literature search was conducted to identify original research articles utilizing ChatGPT in OMS. The Boolean operators “OR” and “AND” were used to refine the search strategy with the following terms: (“ChatGPT” OR “GPT” OR “Chatbot”) AND (“Oral and maxillofacial surgery” OR “oral surgery” OR “maxillofacial surgery”). The search was limited to articles written in English and published up to October 2024. Two independent reviewers (S.-W.O. and B.-E.Y.) conducted the search using the National Library of Medicine (PubMed).

### 2.1. Inclusion and Exclusion Criteria

Studies were included if they were original research articles employing ChatGPT in a structured format containing an introduction, methods, results, and discussion sections [15]. The following types of studies were excluded:

- Non-original research (e.g., review articles, short communications, editorial papers, and scientific briefings);
- Non-English language publications;
- Case reports;
- In vitro and in vivo studies.

### 2.2. Literature Screening and Selection

Following the initial search, two independent reviewers screened the retrieved articles by evaluating their titles and abstracts. Articles meeting the inclusion criteria underwent a full-text review to determine final eligibility. In cases of disagreement between the reviewers, a third reviewer (S.-H.B.) was consulted. Discrepancies were resolved through discussion until a consensus was reached.

## 3. Results

### 3.1. Overview of Included Studies

**Figure 1:**
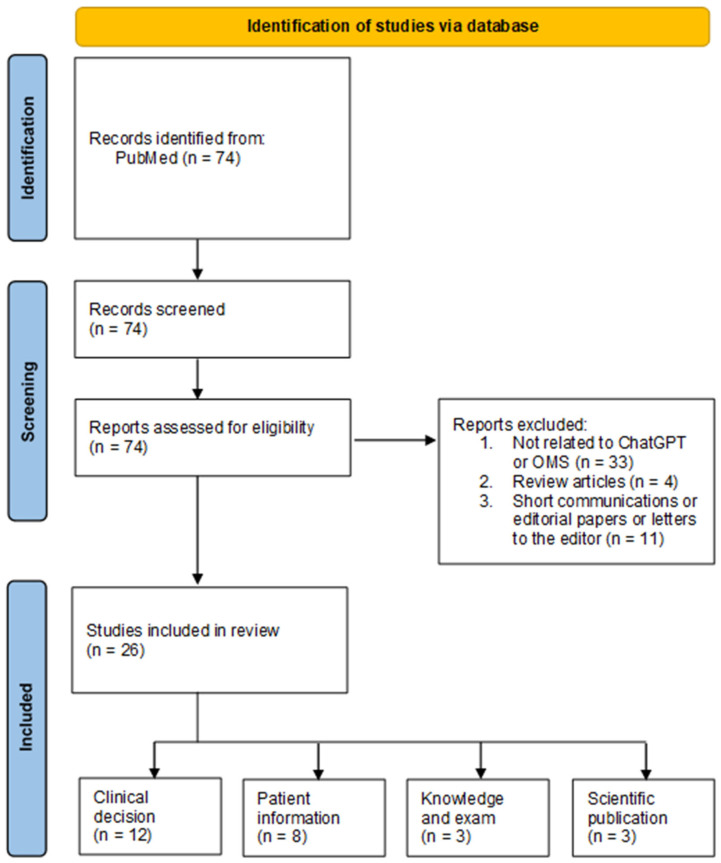
Flow diagram of the paper selection process.

A total of 74 articles were retrieved from PubMed, of which 26 met the inclusion criteria and underwent full-text review. ChatGPT has been applied in various aspects of oral and maxillofacial surgery (OMS), including clinical decision support, patient education, scientific writing, and licensing exam assessments (Figure 1). Each study primarily evaluated ChatGPT’s accuracy for a specific application within OMS, utilizing different language model versions depending on the study period. Additionally, performance comparisons and limitations were frequently discussed (Table 1).

### 3.2. Clinical Decision Support

Among the included studies, 12 articles—the largest category—investigated the role of ChatGPT as a clinical decision support tool in OMS [16,17,18,19,20,21,22,23,24,25,27]. Most studies focused on evaluating the accuracy of ChatGPT’s diagnostic and therapeutic responses in real or simulated clinical scenarios, while a smaller subset assessed its ability to answer knowledge-based or situational judgment questions relevant to patient care (Table 1). Among these, four studies compared the accuracy of ChatGPT’s answers with those of other large language models (LLMs) [17,18,19,27], one study compared responses between different versions of GPT [20], and one study evaluated ScholarGPT (GPT-4-based) against ChatGPT (GPT-3.5-based) [16]. Additionally, one study assessed the differences in clinical management approaches between maxillofacial surgery trainees and ChatGPT [24].

Several studies directly compared ChatGPT’s performance with other LLMs. Frosolini et al. [19] analyzed the triage accuracy of GPT-4-based ChatGPT versus Gemini in 10 real maxillofacial trauma cases. Six oral and maxillofacial surgeons graded the chatbot responses using a Likert scale, revealing that ChatGPT’s recommendations aligned with referral center management in 70% of cases, while Gemini achieved only 50% accuracy. Despite ChatGPT’s superior performance, the authors concluded that both models showed a moderate agreement rate with real-world clinical decisions. Similarly, Lorenzi et al. [18] compared the reliability of treatment recommendations provided by ChatGPT-4 and Gemini Advanced in head and neck malignancy management. Both LLMs produced clinically relevant recommendations, but ChatGPT-4 exhibited superior performance overall.

Rewthamrongsris et al. [17] investigated ChatGPT’s accuracy in infective endocarditis prevention during dental procedures (e.g., tooth extractions and intraoral surgery). They compared seven LLMs against 28 binary clinical questions based on the 2021 American Heart Association guidelines. GPT-4o demonstrated the highest accuracy (80%), followed by Gemini 1.5 Pro (78.57%) and Claude 3 Opus (75.71%). A broader comparison of five chatbot models (GPT-4, GPT-3.5, Bard, Bing, and Claude-Instant) evaluated 50 clinical decision-making questions consisting of multiple-choice and open-ended formats. While no statistically significant differences were observed among the models, GPT-4 demonstrated the highest accuracy [27].

Several studies examined the performance differences between ChatGPT versions. Balel [16] compared GPT-3.5 and ScholarGPT (a GPT-4-based academic model) in answering 60 technical questions on impacted teeth, implants, temporomandibular disorders, and orthognathic surgery. Using a modified Global Quality Scale (GQS), the results indicated that ScholarGPT generated more consistent, high-quality answers than GPT-3.5. Saibene et al. [20] compared GPT-4 and GPT-3.5 in managing five clinical scenarios of odontogenic sinusitis, evaluating the responses based on a total disagreement score (TDS). GPT-4 exhibited significantly lower disagreement scores, indicating greater accuracy and reliability. However, the authors emphasized that newer LLMs still require further validation before they can be fully integrated into evidence-based decision making.

Peters et al. [24] assessed ChatGPT’s performance against maxillofacial surgery trainees in managing 38 patient cases. Three senior maxillofacial surgeons scored responses on the Artificial Intelligence Performance Instrument (AIPI), evaluating differential diagnoses, primary diagnoses, additional examinations, and potential therapeutic approaches. The trainees significantly outperformed ChatGPT (18.71 vs. 16.39, *p* < 0.05). ChatGPT struggled with recommending additional diagnostic tests, reinforcing its current limitations in complex clinical reasoning.

Several additional studies assessed ChatGPT’s accuracy in OMS-related decision making. Işik et al. [21] and Suarez et al. [22] posed 66 and 30 clinical questions, respectively, to ChatGPT and evaluated the responses using a Likert-scale rating. Both studies reported favorable accuracy scores, but lower performance was noted for highly complex queries requiring detailed clinical reasoning.

Vaira et al. [23] conducted a multicenter study analyzing 144 ChatGPT-4-generated responses across 12 OMS subspecialties, evaluating open-ended and closed-ended clinical questions and simulated clinical scenarios. For open-ended questions, accuracy was completely or almost completely correct in 87.2% of cases. For true/false questions, ChatGPT provided correct responses in 84.7% of cases. For clinical scenarios, diagnostic accuracy was 81.7%, but therapeutic recommendations were complete in only 56.7% of cases.

Notably, accuracy varied by subspecialty, with poorer performance in malformative pathology (15.3%), reconstructive surgery (50%), and condylar traumatology (66.7%). A critical limitation identified in Vaira et al.’s [23] study was ChatGPT’s tendency to fabricate references. A total of 46.4% of the bibliographic citations provided by ChatGPT did not exist, illustrating hallucination, a well-documented drawback of LLM-based systems.

### 3.3. Guidance and Information to Patients

Eight studies, the second-largest category among the included literature, assessed ChatGPT’s accuracy in providing patient guidance and information across various OMS subspecialties [28,29,30,31,32,33,34,35]. These studies typically presented common patient inquiries to ChatGPT, collected its responses, and evaluated their accuracy, reliability, and readability.

Balel [32] evaluated ChatGPT’s ability to answer 60 frequently asked patient questions related to impacted teeth, implants, temporomandibular disorders, and orthognathic surgery. Using a modified Global Quality Scale (GQS), 33 experts rated ChatGPT’s responses, which achieved an average score of 4.62 out of 5, indicating high-quality and informative responses. Notably, this study was the earliest research on ChatGPT’s role in patient information within OMS and had the largest number of expert evaluators among the included studies.

Two studies investigated ChatGPT’s accuracy in providing information on third-molar extraction. Jacobs et al. [28] and Aguiar de Sousa et al. [34] presented 25 and 10 common patient questions, respectively, with the responses evaluated by two oral and maxillofacial surgeons. Jacobs et al. [28] compared GPT-3.5’s answers to the American Association of Oral and Maxillofacial Surgeons (AAOMS) consensus paper using a five-point Likert scale. The study reported an average accuracy score of 4.36, suggesting mostly accurate responses with minor omissions or inaccuracies. However, readability analysis revealed that ChatGPT’s answers were overly complex, exceeding the recommended reading level for the average patient. Aguiar de Sousa et al. [34] assessed ChatGPT’s responses to third-molar extraction-related questions sourced via Google Trends analytics. Using the Chatbot Usability Questionnaire, they found that 90.63% of responses were safe and accurate. Despite ChatGPT’s reliability, the authors emphasized that its responses should be validated with appropriate references.

Batool et al. [35] and Cai et al. [33] explored ChatGPT’s ability to answer patient questions on extractions, but with differing approaches. Batool et al. [35] compared responses from an embedded GPT model (custom chatbot based on GPT-3.5-16k) and GPT-3.5 turbo. They evaluated 40 extraction-related patient queries using the Content Validity Index (CVI) with nine expert evaluators on a 4-point Likert scale. The validity scores for OMS-related questions were lower than other dental specialties, with the embedded GPT model scoring 35% and GPT-3.5 turbo scoring 52.5%. The authors attributed this lower performance to the complex nature of OMS topics, requiring deeper contextual understanding. Cai et al. [33] investigated GPT-4’s accuracy in responding to 30 post-operative follow-up questions commonly asked by patients after extractions and other oral surgeries. Three OMS surgeons rated responses using a 0–10 scoring system (higher scores indicating better responses). ChatGPT achieved perfect scores of 10 from all three evaluators, suggesting strong reliability in addressing post-operative patient concerns. A notable finding from this study was that GPT-4 could recognize emotional undertones in patient inquiries and provide empathetic reassurance, a unique feature not previously documented.

Acar [29] conducted the only study directly comparing ChatGPT to other AI chatbots in the context of patient information on dental complications. The study evaluated the effectiveness of GPT-3.5, Bing, and Bard in answering 20 questions about complications following dental implant placement and tooth extraction. Ten OMS surgeons assessed responses using a five-point Likert scale and GQS. ChatGPT consistently achieved the highest scores, followed by Bing and Bard, indicating superior informational quality compared to its competitors.

Coban and Altay [30] assessed ChatGPT’s accuracy in providing information on medication-related osteonecrosis of the jaw (MRONJ). Three OMS surgeons evaluated 120 MRONJ-related questions using the GQS. The average quality score for all responses was 3.9, suggesting moderate to high informational quality. Among question categories, general MRONJ-related queries had the lowest scores, although they were not statistically significant. The authors concluded that while ChatGPT offers patients a fundamental understanding of MRONJ, it may not yet provide comprehensive guidance for complex cases.

A unique study by Manasyan et al. [31] examined ChatGPT’s potential to improve the readability of patient education materials. The study assessed 34 educational documents on alveolar bone grafting for cleft patients, using the Patient Education Material Assessment Tool (PEMAT), Flesch Reading Ease, Flesch–Kincaid Grade Level, and Gunning Fog Index. The results indicated that the average PEMAT score was 67.0, below the recommended threshold of 70%, suggesting that the original materials lacked sufficient quality. Readability analysis showed that the documents were too complex for the average patient, exceeding American Medical Association recommendations. When the materials were rewritten using GPT-3.5, the readability scores significantly improved across all indices, leading the authors to conclude that ChatGPT can enhance the readability of patient education materials without compromising accuracy.

### 3.4. General Knowledge and Exams for OMS

Three studies examined the use of ChatGPT in the field of OMS concerning general knowledge and examinations [10,36,37]. All studies were associated with specific exams, with two evaluating the accuracy of ChatGPT’s responses and one investigating the reliability of automated essay scoring (AES) using ChatGPT for assessing student responses to exam questions.

Morishita et al. [36] evaluated the accuracy of the GPT-4 with vision (GPT-4V) model on questions from the Japanese National Dental Examination, including image-based questions such as X-rays. The study analyzed 160 questions from the 2023 Japanese National Dental Examination, of which 34 were related to oral surgery. GPT-4V’s overall accuracy rate was 35%, with the highest accuracy observed for compulsory questions (57.1%) and the lowest for clinical practical questions (28.6%). The accuracy rate for oral surgery-related questions was 38.2%. Notably, GPT-4V failed to answer 22 of the 160 questions, with 27.3% of unanswered questions related to oral surgery, the second-highest proportion after orthodontics (36.4%). The study also found that the more images included in a question, the greater the likelihood that GPT-4V would fail to generate a response or provide an incorrect answer. The authors concluded that GPT-4V demonstrates limitations in handling image-based and clinical practical questions, suggesting that it is not yet fully suitable as an educational support tool.

Quah et al. [10] evaluated the performance of multiple large language models (LLMs), including GPT-3.5, GPT-4, Llama 2, Gemini, and Copilot, on 259 multiple-choice questions from a previously administered undergraduate OMS examination. The study used a context-aware prompting technique, inputting up to 13 questions at a time into each LLM’s interface. Two faculty members assessed the responses and calculated scores for each model. The average overall score across all models was 62.5%, with GPT-4 achieving the highest score (76.8%), followed by Copilot (72.6%), GPT-3.5 (62.2%), Gemini (58.7%), and Llama 2 (42.5%). By question category, the models performed best in basic science (68.9%) and worst in pharmacology (45.9%). Among the models, Gemini failed to answer 12 questions, Copilot failed to answer 3, and Llama 2 failed on 1, even after three attempts. The authors concluded that LLMs can serve as adjunct tools in medical education but still require further evaluation for reliability and consistency in different subject areas.

Quah et al. [37] compared human-assigned scores with ChatGPT-assigned scores to examine the reliability of AES using ChatGPT in an OMS examination. A total of 69 students participated in an exam consisting of two open-ended questions about infection and trauma. Three authors manually scored the responses, while one author used GPT-4 to perform AES. Each question had a maximum score of 40 points, and the scores assigned by both the human evaluators and ChatGPT were analyzed statistically.

The results showed that the mean manual score for Question 1 (infection) was slightly higher than the score assigned by AES, but the difference was not statistically significant. However, for Question 2 (trauma), the mean manual score was significantly higher than the score assigned by ChatGPT. Further correlation analysis revealed a strong positive correlation between all mean manual scores and AES scores for Question 1, while a moderate positive correlation was observed for Question 2. Interestingly, ChatGPT not only provided numerical scores but also generated concise and structured feedback for each essay response. Despite this advantage, ChatGPT demonstrated a limitation in recognizing essay content that was irrelevant or factually incorrect. The authors concluded that while ChatGPT showed potential for automated essay grading, its tendency to assign lower scores and its inability to identify inappropriate or incorrect content accurately indicate that it is not yet suitable as a standalone tool for assessment or medical education.

### 3.5. Scientific Publication Enhancement

Three studies investigated the use of ChatGPT for scientific publication enhancement in the field of OMS [14,38,39]. These studies focused on whether ChatGPT could assist in generating new manuscript ideas, evaluating research methodology, and providing accurate references.

Balel et al. [14] assessed the idea-generating capability of GPT-4o for systematic reviews in OMS. The study instructed ChatGPT to propose four unpublished systematic review topics each for impacted third molars, implants, orthognathic surgery, and temporomandibular disorders. A literature search was subsequently performed in PubMed to determine whether the suggested topics had already been published. The results showed that 56.25% of the proposed ideas had not yet been published. Among the four categories, the implant-related category had the highest proportion of unpublished ideas at 75%, while the impacted third-molar category had the lowest at 25%. However, the relationship between topic area and originality was not statistically significant. The authors concluded that GPT-4o has the potential to generate novel systematic review topics in OMS but emphasized the need for manual verification of topic originality.

Dang and Hanba [39] investigated ChatGPT’s ability to evaluate the methodology of head and neck oncology research. The study used GPT-3.5 to generate a scoring rubric, which was applied to assess 20 published articles. The results showed that out of the 20 evaluated articles, 8 were rated as “very good,” 9 were rated as “good,” and 3 were rated as “fair.” No articles received the highest grade of “excellent” or the lowest grade of “poor.” Category-specific analysis revealed that the lowest scores were observed in statistical analysis, while the highest scores were in study design and description. Despite the clearly defined scoring criteria, the results showed inconsistencies between different ChatGPT operators, suggesting variability in automated evaluation. The authors concluded that ChatGPT-based methodologies have the potential to improve the peer review process and enhance research transparency, but inconsistencies in scoring must be addressed before widespread implementation.

Wu and Dang [38] examined the accuracy of academic references generated by ChatGPT. The study asked ChatGPT to produce 10 complete references for each category: oral cancer, osteoradionecrosis of the jaw, free-flap reconstruction, adjuvant therapy, and transoral robotic surgery. Two independent evaluators reviewed the references, assessing the accuracy of citation details, including the title, journal, authors, publication year, and digital object identifier (DOI). The study found that only 5 out of 50 references (10%) were completely accurate across all fields. When evaluated by individual citation components, 58% of titles were accurate, DOIs were the least accurate at only 14%, and references for free-flap reconstruction consistently had the lowest accuracy. The authors concluded that ChatGPT exhibits a significant tendency to generate fabricated or inaccurate references, particularly in oral oncology-related research. They emphasized that ChatGPT-generated references should always be verified manually before use in academic writing.

## 4. Discussion

This review aimed to assess the current status of original research utilizing ChatGPT in the field of OMS by analyzing the relevant literature and discussing its limitations and future applications. Among the 26 included studies, the largest proportion focused on clinical decision support (12 studies), followed by guidance and information for patients (8 studies). In contrast, general knowledge and exams (three studies) and scientific publication enhancement (three studies) were the least explored areas.

### 4.1. Discussion of Findings

As a clinical decision support tool, ChatGPT generally demonstrates moderate accuracy, at approximately 70–80% [17,19,22,23]. However, its performance declines when addressing complex differential diagnoses and treatment planning [21,23]. While all reviewed studies acknowledged ChatGPT’s potential, they emphasized that clinicians must exercise caution when interpreting its recommendations, as it remains prone to inaccuracies. Additionally, higher GPT versions tend to provide more accurate responses [20], and ChatGPT appears to outperform other large language models (LLMs) in clinical decision making [17,18,19].

As a tool for patient guidance and education, ChatGPT demonstrates moderate to high accuracy, with some studies reporting up to 90% accuracy [28,33,34]. However, its effectiveness has not been thoroughly examined across all OMS subspecialties. Notably, there is no research evaluating ChatGPT’s accuracy in providing guidance on oral cancer. Furthermore, the limited number of comparative studies makes it unclear whether ChatGPT is superior to other LLMs as a patient information tool.

Regarding general knowledge and examinations, ChatGPT is an adjunct rather than a standalone tool for education and assessment [10,36,37]. The limited number of studies in this domain makes it difficult to draw definitive conclusions, underscoring the need for further research. Additionally, comparative studies between ChatGPT and other LLMs remain scarce, making it challenging to determine whether ChatGPT is the most effective tool.

ChatGPT shows promise in generating research ideas as a tool for scientific publication enhancement, but its effectiveness varies by research topic [14]. While it has demonstrated potential for reviewing research methodology, concerns remain regarding the consistency of its evaluation outcomes [39]. ChatGPT poses a significant risk of generating inaccurate references, necessitating careful verification when used for manuscript writing [38]. Further comparative studies on the role of other LLMs in scientific writing are required. Currently, ChatGPT cannot replace human efforts in research but may assist researchers as a supplementary tool, with the final responsibility for accuracy resting on the user.

ChatGPT has shown potential as a clinical support tool for clinicians and an information provider for patients in OMS. However, its effectiveness as a tool for exams and scientific publication remains suboptimal. ChatGPT should never be used as a standalone tool in clinical practice. Further research is necessary to improve its performance and accuracy, and comparative studies with other LLMs will be essential for determining its future role.

### 4.2. Limitations of AI in Scientific Research and Clinical Practice

As AI technologies, including ChatGPT, continue to advance, they are expected to play an increasingly significant role across various fields. However, several limitations hinder their application in scientific research and healthcare.

One primary concern is hallucination or stochastic parroting, where AI systems generate plausible but inaccurate information [40,41,42]. This includes the potential for fabricated references and incorrect data, raising ethical and legal concerns that could compromise clinical integrity and patient safety [43]. In this review, one study found that ChatGPT provided references with inaccurate paper details [38], while another study reported that 46.4% of its bibliographic references were nonexistent [23].

Another issue is the lack of transparency in AI-generated outputs. The opaque nature of AI algorithms makes it difficult for researchers to understand how conclusions are derived, which can hinder their reproducibility and verification [44]. This lack of transparency may limit trust in AI-generated findings and pose challenges for regulatory approval in healthcare applications.

AI’s inability to replicate human intuition and critical thinking remains a significant limitation [45]. Unlike clinicians, ChatGPT lacks abstract reasoning, ethical judgment, and contextual awareness, which may result in rigid or impersonal recommendations. This limitation raises concerns that AI-generated decisions may not fully account for individual patient contexts, necessitating careful oversight in clinical settings.

### 4.3. Ethical Concerns and Privacy Risks

Privacy-related risks are another major challenge in applying AI-driven chatbots in healthcare. ChatGPT may inadvertently collect and store sensitive patient data, such as medical histories, test results, and diagnoses [46]. There is a risk that this information could be exposed or misused, raising significant concerns about data security and patient confidentiality [47]. Even if de-identified, AI-generated data could be re-identified when combined with other data sources [48].

Among the studies included in this review, those involving real patient data were conducted after obtaining Institutional Review Board (IRB) approval. However, absolute data security cannot be guaranteed, emphasizing the need for stringent safeguards when using AI in clinical research.

### 4.4. Cost and Accessibility Challenges

The financial burden associated with AI technologies also poses a limitation. While ChatGPT is free, premium subscription models such as ChatGPT Plus offer higher efficiency and access to more up-to-date information [49]. Several studies included in this review demonstrated that advanced GPT models outperform free versions in accuracy [10,20,26,27].

Additionally, AI-powered tools are increasingly integrated with third-party applications, potentially incurring premium costs for institutions and researchers [50]. These financial barriers could limit accessibility, particularly in low-resource settings with inadequate electricity, Internet connectivity, or advanced computing infrastructure. Over-reliance on AI-driven decision making could also pose risks if physicians suddenly lose access to these tools, potentially affecting clinical confidence in diagnostics and treatment planning.

### 4.5. Future Research Directions and Areas for Improvement

Upon reviewing original studies utilizing ChatGPT in OMS, several areas for improvement and further investigation have been identified. A key issue is the widespread reliance on Likert scales for evaluating ChatGPT’s accuracy. Likert scales, although widely used in psychometric assessments, generate ordinal data that do not always meet the statistical assumptions required for parametric analysis [51]. This may lead to limitations in statistical power and accuracy, emphasizing the need for alternative evaluation metrics that can more precisely measure ChatGPT’s performance.

There is also a notable lack of research focusing on specific subspecialties. Most existing studies have examined tooth extraction, while areas such as cleft management [31] and medication-related osteonecrosis of the jaw (MRONJ) [30] remain underexplored. Given the high patient demand for preliminary medical information, further research is needed to assess ChatGPT’s accuracy in these domains.

Additionally, scientific publication enhancement and medical exam applications remain under-researched. Despite their potential to improve time efficiency for medical professionals, only three studies have explored these areas in OMS [10,14,36,37,38,39,52]. GPT-powered chatbots could assist in retrieving information from electronic medical records, conducting literature reviews, and improving manuscript formatting [53]. Additional studies are required to evaluate the effectiveness and efficiency of ChatGPT in these applications.

### 4.6. Limitations of This Review

This review has several limitations. As a narrative review, it primarily focused on studies indexed in PubMed, which, while widely recognized in medical and dental research, may not capture all relevant studies. A broader literature search across multiple databases may provide a more comprehensive analysis. Additionally, this review only included literature published in English, raising the possibility of language bias. Future high-quality systematic reviews integrating a wider range of studies will be essential for a more balanced and complete assessment.

## 5. Conclusions

ChatGPT is increasingly being integrated into medicine and dentistry, including OMS. This review highlights its potential benefits in clinical decision support and patient education while underscoring its limitations in accuracy, reliability, and ethical considerations. The findings indicate that research on ChatGPT in OMS is concentrated in specific subspecialties, leaving many critical areas underexplored. Despite its promise, ChatGPT faces significant challenges, including inconsistent accuracy, potential biases, ethical concerns, and the risk of misinformation. These limitations highlight the necessity for rigorous validation studies and continuous technological advancements to improve its reliability and safety. Future research should focus on expanding its evaluation across under-represented OMS areas, refining its integration into clinical workflows, and addressing ethical and regulatory considerations.

In conclusion, ChatGPT should be viewed as an adjunct rather than a replacement for clinical expertise. Oral and maxillofacial surgeons, researchers, and policymakers must collaborate to establish best practices for its implementation, ensuring that AI tools contribute meaningfully to patient care and scientific progress.

## Figures and Tables

**Table 1:** Applications of ChatGPT for original articles in OMS.

Author (Year)	Application Field	Item	GPT	Related Subspecialty	Assessment Tool	Results
Balel (2024) [16]	Clinical decision support	60 questions	Scholar GPT (built on the GPT-4 architecture)	Impacted teeth, dental implants, TMD, and orthognathic surgery	Modified GQS	Scholar GPT > ChatGPT 3.5
Rewthamrongsris et al. (2024) [17]	Clinical decision support	28 questions	ChatGPT ver. 4o	Infection (endocarditis)	The percentage of average accuracy	ChatGPT > Gemini > Claude
Lorenzi et al. (2024) [18]	Clinical decision support	5 questions (cases)	ChatGPT ver. 4	Malignancy	AIPI score	ChatGPT > Gemini advanced
Frosolini et al. (2024) [19]	Clinical decision support	10 cases	ChatGPT ver. 4	Trauma	QAMAI and AIPI scores	ChatGPT > Gemini
Saibene et al. (2024) [20]	Clinical decision support	5 cases (clinical scenario)	ChatGPT ver. 3.5 and 4	Pathology (odontogenic sinusitis)	Total disagreement score	ChatGPT 4 > ChatGPT 3.5
Işik et al. (2024) [21]	Clinical decision support	66 questions	ChatGPT ver. 4 plus	Dental anesthesia, tooth extraction, preoperative and postoperative complications, suturing, writing prescriptions, and temporomandibular joint examination	Likert scale and the modified GQS	The median accuracy score was 5, and the median scores of hard-level questions were found to be lower.
Suarez et al. (2024) [22]	Clinical decision support	30 questions	ChatGPT ver. 4	Pathology, oncology, third-molar extraction, and periapical surgery	Likert scale	The overall accuracy: 71.7%
Vaira et al. (2024) [23]	Clinical decision support	72 open-ended questions, 72 closed-ended questions, and 15 clinical scenarios	ChatGPT ver. 4	Pathology, oncology, reconstruction, orthognathic surgery, TMD, and trauma	Likert scale	AI’s ability to resolve complex clinical scenarios is promising, but it still falls short of being considered a reliable support for the decision-making process.
Peters et al. (2024) [24]	Clinical decision support	4 questions associated with clinical cases	ChatGPT ver. 3.5	Infection, trauma, pathology, TMD, and oncology	AIPI score	ChatGPT < trainee
Uranbey et al. (2024) [25]	Clinical decision support	2 questions (diagnosis and treatment)	ChatGPT ver. 3.5	Pathology and oncology	Likert scale	ChatGPT exhibited high accuracy in providing differential diagnoses and acceptable treatment plans.
Lee et al. (2024) [26]	Clinical decision support	Mandibular anteroposterior position	ChatGPT ver. 3.5 and 4	Dentofacial deformity	Balanced accuracy and F1-score	By converting cephalometric measurements into intuitive text formats, LLMs significantly enhanced the accessibility and clinical interpretability of diagnostic processes.
Azadi et al. (2024) [27]	Clinical decision support	50 questions (open ended and multiple choice)	ChatGPT ver. 3.5 and 4	Trauma, pathology, orthognathic surgery, and implants	GQS	No significant differences among different chatbots
Jacobs et al. (2024) [28]	Patient information	25 questions	ChatGPT ver. 3.5	Third-molar extraction	Likert scale	Most responses were accurate, with minor inaccuracies or missing information.
Acar (2023) [29]	Patient information	20 questions	ChatGPT ver. 3.5	Dental implant and tooth extraction	Likert scale and GQS	ChatGPT > Bing > Bard
Coban and Altay (2024) [30]	Patient information	120 questions	ChatGPT ver. 3	Pathology (MRONJ)	GQS	ChatGPT showed moderate quality to questions about MRONJ.
Manasyan et al. (2024) [31]	Patient information	34 patient education materials	ChatGPT ver. 3.5	Clefts	Flesch Reading Ease, Flesch–Kincaid Grade Level, and Gunning Fog Index	AI rewriting significantly improved the readability among all assessed metrics.
Balel (2023) [32]	Patient information	60 patient questions and 60 technical questions	Not indicated	Impacted teeth, dental implants, TMD, and orthognathic surgery	Modified GQS	ChatGPT has significant potential as a tool for patient information in oral and maxillofacial surgery. However, its use in training may not be completely safe at present.
Cai et al. (2024) [33]	Patient information	30 questions	ChatGPT ver. 4	Tooth extraction and pathology	Score evaluated by experts	ChatGPT/GPT-4 could be used for patient follow-up after oral surgeries with careful consideration of limitations and under the guidance of healthcare professionals.
Aguiar de Sousa et al. (2024) [34]	Patient information	10 questions	Not indicated	Third-molar surgery	CUQ	ChatGPT offers accurate and scientifically backed answers (CUQ: 90.63%).
Batool et al. (2024) [35]	Patient information	10 questions	ChatGPT ver. 3.5 turbo	Tooth extraction	Likert scale	Embedded ChatGPT > ChatGPT
Quah et al. (2024) [10]	Knowledge and exam	259 questions (multiple choice)	ChatGPT ver. 3.5 and 4	General oral surgery	The mean overall score	GPT-4 > Copilot > GPT-3.5 > Gemini > Llama 2
Morishita et al. (2024) [36]	Knowledge and exam	160 questions (the number of OMS questions were not indicated)	ChatGPT ver. 4 with vision	General oral surgery	The percentage of correct answers	Overall rate of 35.0% (OMS: 38.2%)
Quah et al. (2024) [37]	Knowledge and exam	2 questions (essay)	ChatGPT ver. 4	Infection and trauma	Automated essay scoring	Positive correlations between ChatGPT and manual essay scoring
Balel et al. (2024) [14]	Scientific publication enhancement	16 unpublished systematic review ideas	ChatGPT ver. 4o	Impacted teeth, dental implants, TMD, and orthognathic surgery	Percentage of ideas not searched on PubMed	56.25% (9/16) of ideas were not found in the PubMed database.
Wu and Dang (2023) [38]	Scientific publication enhancement	50 references from 5 commonly researched keywords	Not indicated	Oncology and reconstruction	A numerical score	Only 10% of the articles provided by ChatGPT were correct regarding head and neck surgery.
Dang and Hanba (2024) [39]	Scientific publication enhancement	20 articles	ChatGPT ver. 3.5	Malignancy	A scoring system generated by ChatGPT	The preliminary feasibility of ChatGPT in assessing the methods sections was demonstrated.

GPT, Generative Pre-Trained Transformer; TMD, temporomandibular disorder; GQS, Global Quality Scale; AIPI, Artificial Intelligence Performance Instrument; QAMAI, Quality Analysis of Medical Artificial Intelligence; AI, artificial intelligence; LLMs, large language models; MRONJ, medication-related osteonecrosis of the jaw; CUQ, Chatbot Usability Questionnaire; OMS, oral and maxillofacial surgery.

## Data Availability

The data presented in this study are available on request from the corresponding author.
